# The Impact of *Flt3* Gene Mutations in Acute Promyelocytic Leukemia: A Meta-Analysis

**DOI:** 10.3390/cancers11091311

**Published:** 2019-09-05

**Authors:** Gledson L. Picharski, Diancarlos P. Andrade, Ana Luiza M. R. Fabro, Luana Lenzi, Fernanda S. Tonin, Raul C. Ribeiro, Bonald C. Figueiredo

**Affiliations:** 1 Instituto de Pesquisa Pelé Pequeno Príncipe, 1532 Silva Jardim, AV., Curitiba, Paraná 80250-200, Brazil; 2 Faculdades Pequeno Príncipe, 333 Iguaçu Av., Rebouças, Curitiba, Paraná 80230-902, Brazil; 3 Unidade de Hematologia e Oncologia Pequeno Príncipe Hospital, 1070 Dsembargador Motta Av., Curitiba, Paraná 80250-060, Brazil; 4 Universidade Federal do Paraná, 632 Pref Lothário Meissner Av., Curitiba, Paraná 80210-170, Brazil; 5 Department of Oncology, Leukemia and Lymphoma Division, St. Jude Children’s Research Hospital, 262 Danny Thomas Place, Memphis, TN 38105, USA; 6 Centro de Genética Molecular e Pesquisa do Câncer em Crianças (CEGEMPAC), 400 Agostinho Leão Jr. Av., Curitiba, Paraná 80030-110, Brazil; 7 Departamento de Saúde Coletiva, Universidade Federal do Paraná, 260 Padre Camargo St., Centro, Curitiba, Paraná 80060-240, Brazil

**Keywords:** acute promyelocytic leukemia, APL, *FLT3*-ITD, *FLT3*-D835, WBC

## Abstract

The association of *FLT3* mutations with white blood cell (WBC) counts at diagnosis and early death was studied in patients with acute promyelocytic leukemia (APL). Publications indexed in databases of biomedical literature were analyzed. Potential publication bias was evaluated by analyzing the standard error in funnel plots using the estimated relative risk (RR). Mixed-effect models were used to obtain the consolidated RR. All analyses were conducted using the R statistical software package. We used 24 publications in the final meta-analysis. Of 1005 males and 1376 females included in these 24 publications, 645 had *FLT3*-ITD (internal tandem duplication) mutations. Information on *FLT3*-D835 mutations was available in 10 publications for 175 patients. Concurrent occurrence of the two mutations was rare. WBC count at diagnosis was ≥10 × 10^9^/L in 351 patients. For patients with the *FLT3*-ITD mutation, RR was 0.59 for overall survival (OS) and 1.62 for death during induction. For those with *FLT3*-D835 mutations, the RR was 0.50 for OS and 1.77 for death during induction. RR for WBC count ≥10 × 10^9^/L was 3.29 and 1.48 for patients with *FLT3*-ITD and *FLT3*-D835, respectively. APL patients with *FLT3*-ITD or *FLT3*-D835 are more likely to present with elevated WBC counts and poorer prognosis than those without these mutations.

## 1. Introduction

Acute promyelocytic leukemia (APL) is a unique subtype of acute myeloid leukemia (AML) characterized by coagulopathy and the accumulation of morphologically aberrant promyelocytes carrying one of the rearrangements involving the *RARAα* gene, which encodes the retinoic acid receptor alpha located at 17q21. Among these, the rearrangement of *RARAα* with the promyelocytic leukemia (*PML*) gene in the translocation t(15;17)(q22;q12) occurs in approximately 95% of APL patients [1]. The frequency of APL varies by ethnic group, and APL accounts for 10–25% of all AML cases [2]. Although RARAα fusion proteins are required for APL leukemogenesis, additional genetic aberrations such as mutations in *FLT3*, which encodes FMS-like tyrosine kinase 3, are frequently found in APL and might contribute to its pathogenesis and outcome [3]. Two common types of *FLT3* aberrations have been considered clinically relevant. The most common *FLT3* aberration is an internal tandem duplication (ITD) that results in constitutive activation of the tyrosine kinase receptor. *FLT3*-ITD occurs in 13–40% of APL patients [4,5]. The other *FLT3* aberration is a point mutation in the region encoding the activation loop, usually in the codon for aspartic acid 835 (D835), which occurs in approximately 8% of APL patients. Worse outcomes have been reported for patients harboring either of these mutation types [6]. The prognostic significance of *FLT3*-D835 mutations in non-promyelocytic AML was evaluated in a previous meta-analysis including only adult patients [7]. These authors concluded that the patients with *FLT3*-D835 mutations with intermediate cytogenetics had similar overall survival as wild-type *FLT3* patients, and adult AML patients with *FLT3*-D835 mutations exhibited better outcomes than those with *FLT3*-ITD.

The management of APL has dramatically improved over the past decade [8]. Patients with WBC count less than 10 × 10^9^/L at diagnosis are currently treated with a combination of all-*trans* retinoic acid (ATRA) and arsenic trioxide (ATO), which are associated with survival rates or more than 90% [9]. For other patients, it is possible to achieve similar survival rates by adding an anthracycline to the ATRA–ATO regimen [10,11]; however, early mortality remains high [12]. Identifying patients at high risk of early death or having resistant genotypes can aid the development and implementation of alternative strategies for adapted management. The association of *FLT3*-ITD and *FLT3*-D385 mutations with white blood cell (WBC) counts at diagnosis and early death has not been systematically evaluated in APL patients. Therefore, we performed a systematic review with meta-analysis of publications reporting data on *FLT3*-ITD and/or *FLT3*-D835 mutations, WBC counts, early mortality rates, and outcomes of APL patients.

## 2. Results

### 2.1. Search Results

**Figure 1:**
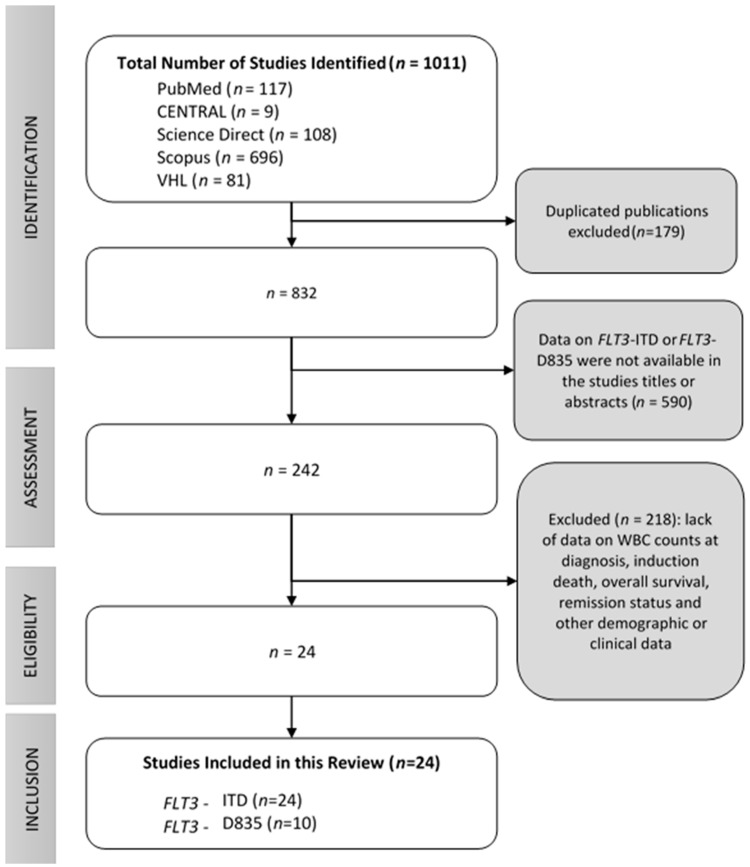
Flowchart to identify studies on acute promyelocytic leukemia with the acquired mutations *FLT3*-ITD (internal tandem duplication) and *FLT3*-D835.

In the systematic searchers, we identified 832 articles after duplicates removal that were screened by titles and abstracts. Of these, 590 were excluded because they were considered irrelevant to the research. Evaluation of the full text was performed for 242 studies, of which 218 were excluded given the lack of outcome or mutations of interest. Finally, a total of 24 articles were included in this systematic review [13,14,15,16,17,18,19,20,21,22,23,24,25,26,27,28,29,30,31,32,33,34,35,36], as shown in Figure 1.

All 24 publications reported data on the *FLT3*-ITD mutation and included a total of 2381 APL patients. Of these 2381 patients, 645 (27.1%) had the *FLT3*-ITD mutation. Only 10 of the included publications reported data on the *FLT3*-D835 mutation, they and included a total of 1104 APL patients. Of these 1104 patients, 175 (15.8%) had the *FLT3*-D835 mutation. The *FLT3*-ITD and *FLT3*-D835 mutations co-occurred in 24 patients. Table 1 presents details of patient numbers, demographics, clinical features, and mutation type for each study.

**Figure 2:**
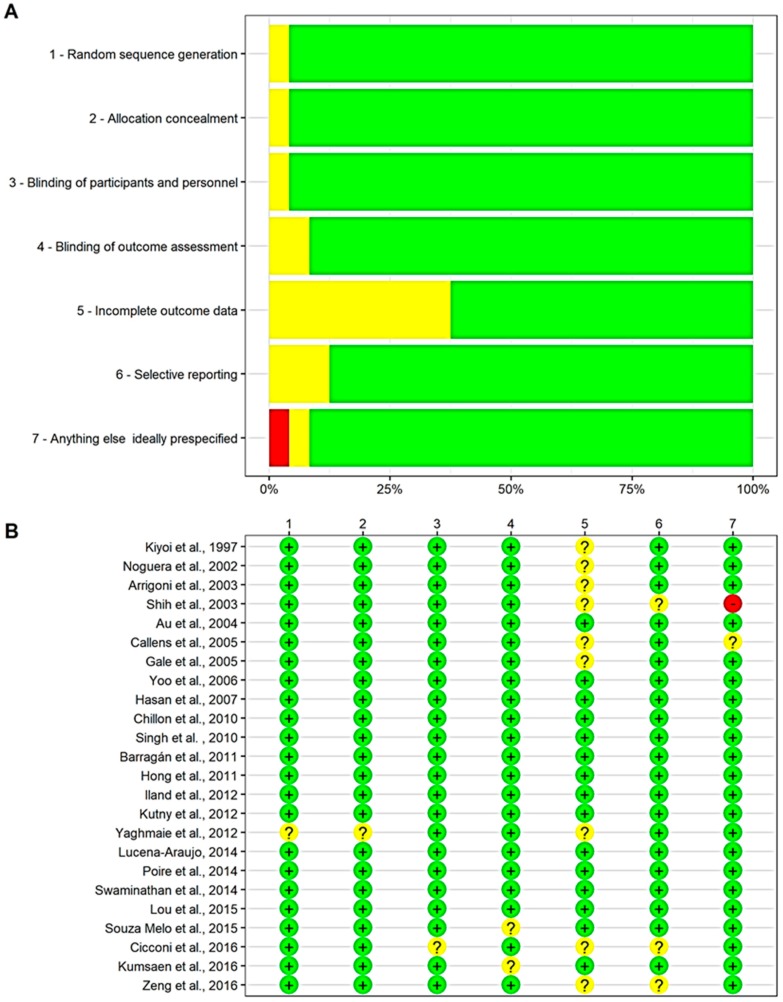
Risk of bias evaluation of each included study. (**A**) Risk of bias graph; (**B**) Risk of bias summary.

Overall, studies presented a low risk of bias for all the domains, according to the risk of bias assessment tool (Figure 2). Incomplete outcome data was unclear in around 40% of studies, while selective reporting was unclear in less than 10%. Only one study presented a high risk of bias for other factors. The methodological quality was further evaluated by constructing funnel plots using relative risk data, which ensured that the effect of publication bias on the overall study results was minimized. The methodological quality of publications included in this study was assessed by using the seven Cochrane criteria for risk of publication bias, and the risk of bias was designated as high, unclear, or low (Figure 2). The methodological quality was further evaluated by constructing funnel plots using relative risk (RR) data, which minimized the effect of publication bias on the overall study results.

### 2.2. Sex and WBC Count According to FLT3 Status

**Figure 3:**
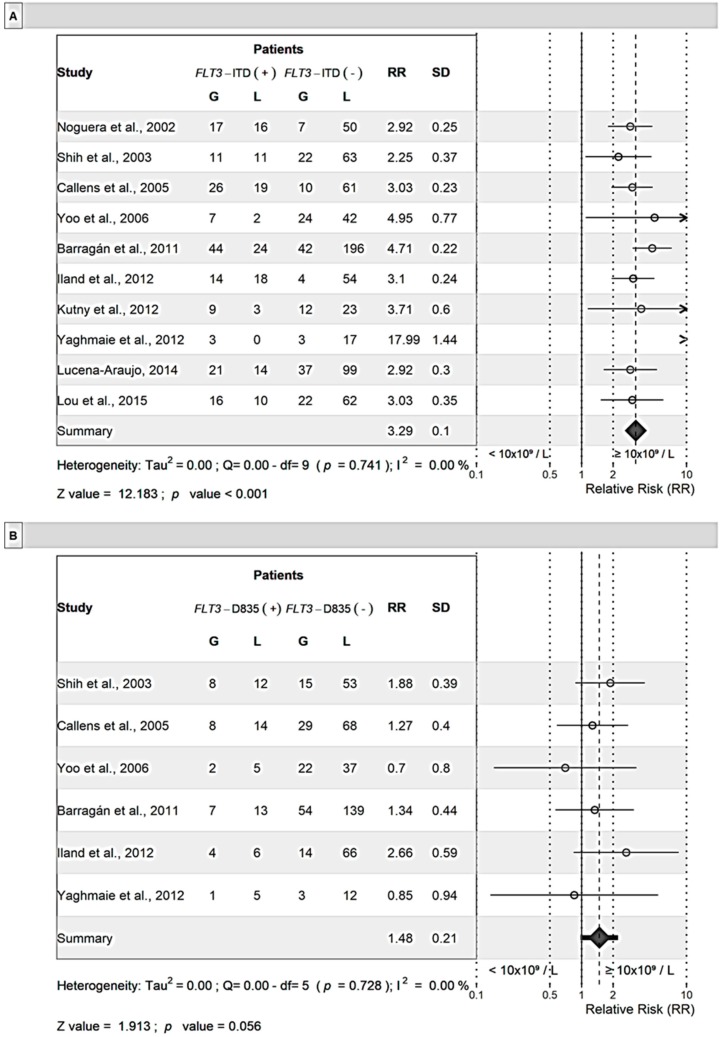
Meta-analysis of the relative risk (RR) by white blood cell count for *FLT3*-ITD (**A**) and *FLT3*-D835 (**B**) mutations. Mixed effect models were used for analysis. G: white blood cell count equal or greater than 10 × 10^9^/L and L lower than 10 × 10^9^/L. The numbers in each column represent the number of cases (patients).

Figure 3 shows RR data of each study for *FLT3*-ITD and *FLT3*-D835 mutations by WBC count (≥10 × 10^9^/L or <10 × 10^9^/L). There was no difference in the RR of *FLT3*-ITD mutations by sex. However, patients with this mutation were at an elevated risk (RR = 3.29; *p* < 0.001) of having WBC counts ≥10 × 10^9^/L. For patients with *FLT3*-D835 mutations, the sample size was small in terms of the number of publications as well as the number of patients included in risk assessment analysis. Although the RR was not significantly different from 1, a slightly higher RR was observed for male patients (RR = 1.23, *p* = 0.314) than for female patients, and those with WBC count ≥ 10 × 10^9^/L (RR = 1.48, *p* = 0.056) vs. WBC count <10 × 10^9^/L.

### 2.3. Outcome According to FLT3 Status

**Figure 4:**
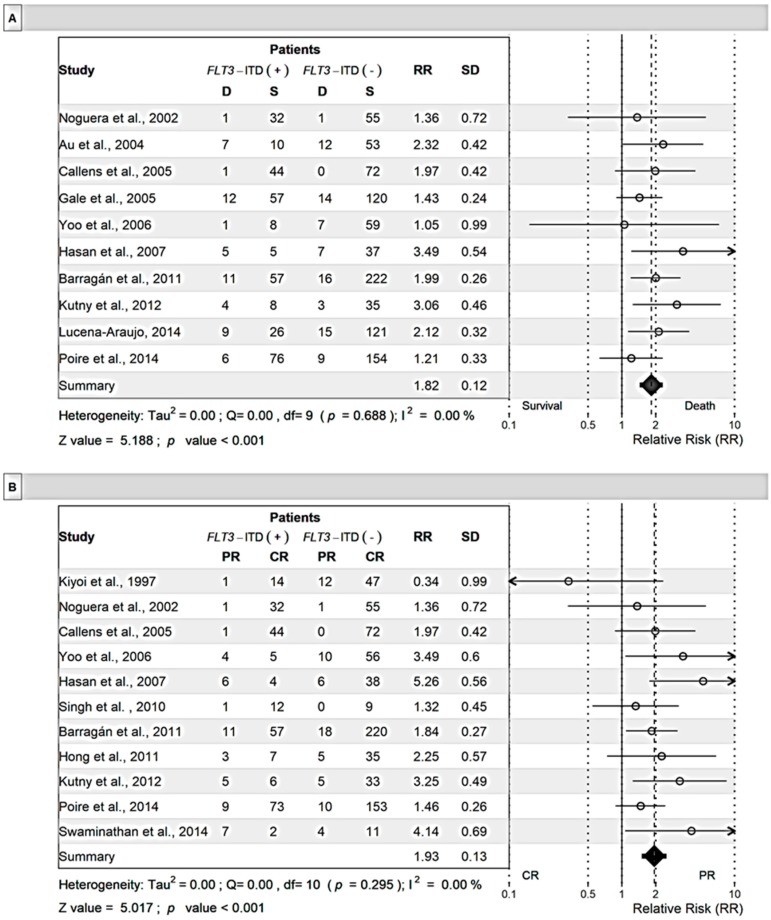

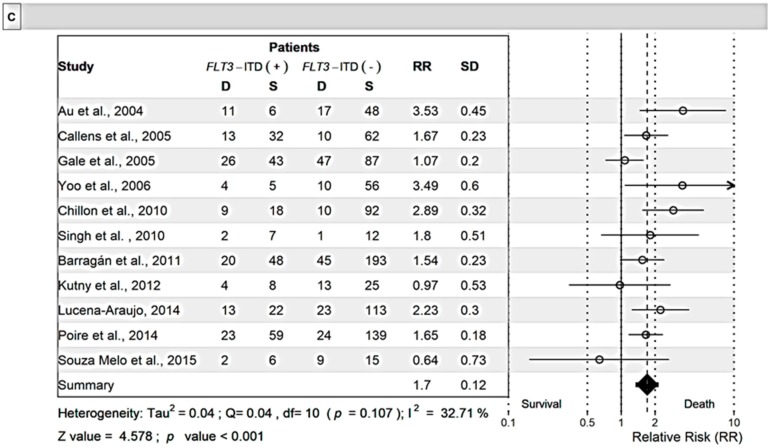
Meta-analysis of the relative risk for patients with the *FLT3*-ITD mutation. Induction death (**A**), complete remission (**B**), and overall survival (**C**). Mixed effects models were used for analysis. D: Deceased patients; S: Surviving patients; R: Complete Remission; and PR: Partial Remission. Cases positive or negative for *FLT3*-ITD or *FLT3*-D835 were divided into deceased patients and surviving patients. The numbers in each column represent the number of cases (patients).

The RR for death during the induction phase was 1.82-fold (*p* < 0.001) higher in patients with the *FLT3*-ITD mutation than those without this mutation (Figure 4). For this measure, RR values reported in the included publications were between 1.05 and 3.49. Patients with the *FLT3*-ITD mutation were also less likely to attain complete remission (CR) (RR = 0.59, *p* = 0.003) than those without this mutation. Patients with the *FLT3*-ITD mutation also had lower overall survival (OS) rates (RR = 0.59, *p* < 0.001) and a higher likelihood of dying of any cause (1.70-fold) than those without this mutation. 

**Figure 5:**
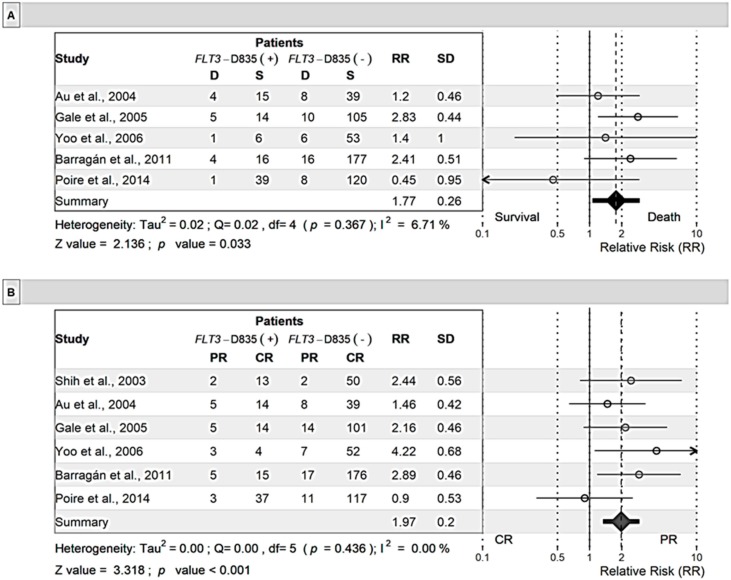

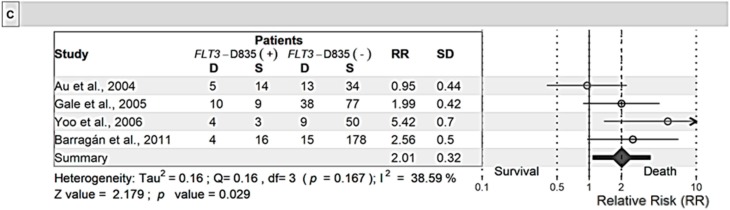
Meta-analysis of the relative risk for patients with the *FLT3*-D835 mutation. Induction death (**A**), complete remission (**B**), and overall survival (**C**). Mixed effects models were used for analysis. D: Deceased patients; S: Surviving patients; R: Complete Remission; and PR: Partial Remission. Cases positive or negative for *FLT3*-ITD or *FLT3*-D835 were divided into deceased patients and surviving patients. The numbers in each column represent the number of cases (patients).

Also, death during induction was more frequent in patients with the *FLT3*-D835 mutation than those with wild-type (WT) *FLT3* (RR = 1.77; *p* = 0.033; Figure 5). Consistent with this observation, the proportion of patients with *FLT3*-D835 attaining remission was lower than that of patients with WT *FLT3* (RR = 0.55, *p* < 0.001); there was a 1.82-fold higher likelihood of CR in patients without this mutation than in patients with this mutation. Presence of the *FLT3*-D835 mutation also doubled the risk of death (RR of survival = 0.5; *p* = 0.029). It is important to note that the sample size of patients with the *FLT3*-D835 mutation was small, which increased the variability of data.

## 3. Discussion 

Outcomes of patients with the *FLT3*-ITD mutation were significantly worse than those without this mutation. Remarkably, high WBC count ≥10 × 10^9^/L was associated with poor prognosis, probably because *FLT3* mutations are more likely associated with higher WBC. During the induction phase, death rates were significantly higher for patients with the *FLT3*-ITD mutation than those without this mutation. In only 3 of the 24 publications [27,31,37], CR rates were comparable for those with or without *FLT3*-ITD mutations. In the remaining 19 publications, CR rates were lower in patients with *FLT3*-ITD mutations than those without *FLT3*-ITD. Similarly, the five-year OS rates were lower in patients with *FLT3*-ITD mutations than those without *FLT3*-ITD mutations. Thus, our analysis confirmed that patients with these mutations were less likely to achieve remission and more likely to die of any cause. These results are consistent with those from single-arm studies analyzing the clinical impact of *FLT3*-ITD mutations in APL [14,15,16,24,26,27,38,39]. Moreover, our findings agree with those reported in a meta-analysis on 11 publications analyzing the RR of event-free survival and OS [6]. Compared with this, our study included more publications and analyzed the RR in relation to more variables.

Data analysis on the *FLT3*-D835 mutation was limited due to low frequency of reporting on this mutation and high variability of results among publications. Nonetheless, all publications included in our analyses indicated a worse outcome in patients with the *FLT3*-D835 mutation than in those without this mutation. During the induction phase, death was more likely to occur in patients with the *FLT3*-D835 mutation than in those without this mutation, resulting in lower CR rates in the former group. OS rates were reported in only five publications and our meta-analysis revealed a broad confidence interval (CI); however, there was a two-fold decrease in the likelihood of survival in patients with the *FLT3*-D835 mutation than those without this acquired mutation.

Among presenting features, only WBC count at diagnosis was different between those with or without *FLT3* mutations. Patients with APL harboring *FLT3*-ITD had a significantly higher WBC count at presentation than those without this mutation. However, data are less compelling for APL patients carrying the *FLT3*-D835 mutation: RR values included values smaller than 1, and the *p*-value was only marginally significant (*p* = 0.056). The reduced OS associated with *FLT3*-D835 mutations in APL patients demonstrated in the present study was not previously confirmed among non-promyelocytic AML [7], suggesting that these mutations play different roles in these leukemia subtypes. Thus, APL patients with the *FLT3*-D835 mutation also showed a worse prognosis compared to APL patients with wild-type *FLT3*. However, the number of studies including information on the *FLT3-D835* status is relatively small. 

The prognostic implications of *FTL3*-ITD, in general, continue to be uncertain because of the small number of failures in current protocols. However, recent articles suggest synergist cytotoxicity of the combination of arsenic and ATRA but not of the combination of ATRA and standard chemotherapy for cases with APL and *FLT3*-ITD [18,40]. The European Leukemia Net expert panel has provided important insights about the recent advances in the management of APL [8]. These guidelines contain specific recommendations for optimizing treatment in high-risk disease, but do not include specific recommendations for patients with *FLT3* mutations.

Patients with non-APL myeloid leukemia with *FLT3*-ITD mutations have a poor prognosis. Their outcomes did not improve even with the use of *FTL3* inhibitors and hematopoietic stem-cell transplantation [41]. Despite promising efficacies of FLT3 inhibitors in aggressive AML with and without *FLT3* mutations, the outcome is still reserved because of rapid development of resistance. Therefore, next-generation FLT3 inhibitors are awaited, probably associated with other targeted agents [42,43]. While enthusiasm to use *FLT3* inhibitors in APL has not yet reached satisfactory results, arsenic in combination with ATRA overcomes the implications of *FLT3* mutations in APL. 

However, outcomes of APL patients with *FLT3*-ITD mutations appear to be improving with changes in management. A recent study of APL patients harboring *FLT3*-ITD revealed that those randomized to receive ATO had a significantly better prognosis than those who did not receive ATO [40]. Management of leukocytosis at diagnosis in APL patients, including the use of prophylactic steroids, has reduced early death rates [41]. Given these findings, we suggest that the introduction of ATO for all patients with newly diagnosed APL and strict compliance with guidelines for the initial management of APL, particularly those with hyperleukocytosis, can help eliminate the prognostic value of *FTL3* mutations in APL. Latin-American countries having increased frequency of patients with *FLT3*-ITD and advanced disease [44] might be most benefited by this approach [18,45]. 

Our study has some limitations. Although meta-analysis is an analytical technique designed to summarize the results of multiple studies, the inappropriate combination of data can generate misleading conclusions. The impossibility of collecting uniform data on white blood cell counts (e.g., some studies reported only the median and other studies reported this data using different metrics) hampered some meta-analyses. Similarly, reporting of the adverse events of interest was not uniform in primary studies. In some reports, actual numbers were included while in others only the percentage. In the latter cases, it was necessary to calculate the absolute number. We tried to minimize this type of bias by calculating further values of raw data and by evaluating between-trial heterogeneity and publication bias (sensitivity analyses). Poor quality of included studies could also negatively impact the meta-analysis results; however, in our study we did not find critical issues in primary studies that could bias our conclusions.

## 4. Methods

### 4.1. Protocol

The protocol for this systematic review with meta-analysis was generated according to the PRISMA guideline and using the International Prospective Register of Systematic Reviews (PROSPERO), an open-access database of systematic reviews (2015: CRD42015025752b).

### 4.2. Search Strategy and Eligibility Criteria 

An extensive literature search was performed in PubMed, Cochrane Central Register of Controlled Trials (CENTRAL), Science Direct, Scopus, and Virtual Health Library (VHL) databases. Search strategies were customized to each scientific database (Tables S1–S6). We included interventional studies (randomized or non-randomized clinical trials) published in English, Spanish, or Portuguese up to June 2019 for which the data could be extracted directly or by calculation using published information. Studies were included when assessing patients of any age or gender diagnosed with APL, also referred to as acute myeloid leukemia (AML) FAB M3; exposure reported as acquired mutations, secondary mutations, or somatic mutations (*FLT3*-ITD or *FLT3*-D835); and outcomes/events of interest reported as mortality, early death, survival, or prognosis. Studies evaluating other types of leukemia or not reporting mutations information and other types of studies (e.g., review, meta-analysis, comments, editorials, letters, case reports) were excluded. 

### 4.3. Selection of Publications, Data Extraction, and Quality Assessment

Selection of publications, data extraction, and quality assessment were performed by two researchers (GLP and DPA) who conducted each part of the process independently. The researchers met to assess divergences at each stage of the analysis. A list of identified clinical trials was prepared, and each researcher independently decided to exclude or include a publication after evaluating the title and abstract. Disagreements were jointly examined and resolved. 

The following data were extracted from each publication: author(s), title, year of publication, number of patients, *FLT3* mutational status (wild-type (WT), *FLT3*-ITD, D385), sex, WBC counts ≥10 × 10^9^/L or <10 × 10^9^/L, death during the induction phase (early death), and outcome (complete remission (CR), partial remission (PR), and overall survival (OS) rates). All data were assessed by using the Cochrane risk-of-bias criteria for quality assessment [46]. Differences in assessments between the two researchers were consensually resolved.

### 4.4. Outcome Measures and Statistical Analyses

For the meta-analysis, we included studies for which data could be calculated as effect-size measures (i.e., studies properly reporting outcome results). For each mutation, the following variables were considered: sex; WBC ≥10 × 10^9^/L or <10 × 10^9^/L; death during the induction phase; and PR, CR, and five-year survival rates. Data on patients having either the *FLT3*-ITD or *FLT3*-D835 mutation and those having co-occurrence of mutations were analyzed separately. The statistical program R was used for all analyses [47]. The metafor package, which has a comprehensive collection of functions required for conducting a meta-analysis in R, was used [48,49]. 

The RR for each outcome measure reported in individual publications was used in the meta-analysis. In all cases, mixed-effects models were used [50], with a 95% confidence interval (CI) and a restricted maximum likelihood estimation to obtain the consolidated RR. The X² and I² tests were used to evaluate the heterogeneity among publications, which was classified by using I² values as follows: none (I² close to 0%), low (I² close to 25%), moderate (I² close to 50%), or high (I² close to 75%) [51]. If moderate or high heterogeneity was observed, models were re-evaluated and the effect of withdrawing the relevant publications was analyzed. Potential publication bias was evaluated by analyzing the standard error in funnel plots by using the estimated RR [50,52].

## 5. Conclusions

Our study confirms that patients with *FLT3*-ITD mutations are more likely to have significantly higher WBC counts at diagnosis, higher risk of induction deaths, and lower OS rates than those without *FLT3*-ITD mutations. The *FLT3*-D835 mutation was also associated with elevated WBC counts, although they were lower than those associated with the *FLT3*-ITD mutation. However, the prognostic implications of the *FLT3*-D835 mutation remain to be determined in future studies. 

## Figures and Tables

**Table 1:** Features of the studies included in this systematic review.

Study [Reference]	*n*	Male *n* (%)	WBC ≥ 10 × 10^9^/L *n* (%)	Early Death *n* (%)	*FLT3*-ITD *n* (%)	*FLT3*-D835 *n* (%)
Arrigoni, 2003 [13]	29	16 (55.2%)	-	-	10 (34.5%)	-
Au, 2004 [14]	82	39 (47.6%)	-	19 (23.2%)	17 (20.7%)	19 (23.2%)
Barragán, 2011 [15]	306	155 (50.7%)	86 (28.1%)	27 (8.8%)	68 (22.2%)	20 (9.4%)
Callens, 2005 [16]	117	54 (46.2%)	36 (30.8%)	1 (0.9%)	45 (38.5%)	22 (19.6%)
Chillón, 2010 [17]	129	-	-	-	27 (20.9%)	12 (9.3%)
Cicconi, 2016 [18]	159	81 (50.9%)	-	-	33 (20.8%)	
Gale, 2005 [19]	203	-	-	26 (12.8%)	69(34.0%)	19 (14.2%)
Hasan, 2007 [20]	54	30 (55.6%)	-	12 (22.2%)	10 (18.5%)	
Hong, 2011 [21]	50	22 (44.0%)	-	-	10 (20.0%)	
Iland, 2012 [22]	90	-	18 (20.0%)	-	32 (35.6%)	10 (11.1%)
Kiyoi, 1997 [23]	74	39 (52.7%)	-	-	15 (20.3%)	-
Kutny, 2012 [24]	50	32 (64.0%)	21 (42.0%)	7 (14.0%)	12 (24.0%)	-
Lou, 2015 [25]	110	61 (55.5%)	38 (34.5%)	-	26 (23.6%)	-
Lucena, 2014 [26]	171	85 (49.7%)	58 (33.9%)	24 (14.0%)	35 (20.5%)	-
Noguera, 2002 [27]	90	43 (47.8%)	24 (26.7%)	2 (2.2%)	33 (36.7%)	-
Poiré, 2014 [28]	245	134 (54.7%)	-	15 (6.1%)	82 (33.5%)	40 (23.8%)
Shih, 2003 [29]	107	50 (46.7%)	33 (30.8%)	-	22 (20.6%)	20 (22.7%)
Singh, 2010 [30]	22	11 (50.0%)	-	-	9 (40.9%)	-
Yoo, 2006 [31]	75	34 (45.3%)	31 (41.3%)	8 (10.7%)	9 (12.0%)	7 (10.4%)
Souza, 2015 [32]	34	18 (52.9%)	-	-	8 (23.5%)	-
Swaminathan, 2014 [33]	40	21 (52.5%)	-	-	10 (25.0%)	-
Yaghmaie, 2012 [34]	23	19 (82.6%)	6 (26.1%)	-	3 (13.0%)	6 (28.6%)
Zeng, 2016 [35]	69	33 (48%)	-	-	50 (72%)	-
Kumsaen, 2016 [36]	52	28 (54%)	-	-	10 (19%)	-
Total	2381	1005	351	141	645	175

WBC: white blood cells count; Early death: death during the induction phase; *FLT3*-ITD %: percentage of mutation; *FLT3*-D835 %: percentage of mutation.

## Supplementary Materials

The following are available online at https://www.mdpi.com/2072-6694/11/9/1311/s1, Table S1: Keyword structure and conceptual logic of the search strategy for each search engine, Table S2: Search terms used for the PubMed database, Table S3: Search terms used for the Cochrane Central Register of Controlled Trials (CENTRAL) database, Table S4: Search terms used for the Science Direct database, Table S5: Search terms used to query the Scopus database, Table S6: Search terms used for the Virtual Health Library (VHL) database.
