# Supplementary material for: The Impact of Flt3 Gene Mutations in Acute Promyelocytic Leukemia: A Meta-Analysis
Source: Cancers (Basel). 2019 Sep 5;11(9):1311. doi: 10.3390/cancers11091311 (PMC6770268; doi:10.3390/cancers11091311)
Supplement: Supplementary file 1 [file cancers-11-01311-s001.pdf]

Supplementary Materials: The Impact of Flt3 Gene Mutations in Acute Promyelocytic Leukemia: A Meta-Analysis

Gledson Luiz Picharski, Diancarlos Pereira de Andrade, Ana Luiza de Melo Rodrigues Fabro, Luana Lenzi, Fernanda S. Tonin, Raul C. Ribeiro and Bonald C. Figueiredo

Table S1. Keyword structure and conceptual logic of the search strategy for each search engine.

Element	Search Keyword
Patients	"Acute Promyelocytic Leukemia," "Acute Myeloid Leukemia FAB M3," "AML M3."
Exposure	Acquired mutations, secondary mutations, somatic mutations, somatic changes, *FLT3*, internal tandem duplications, ITD, D835, *RAS*, TKD, "tyrosine kinase," TKI, *NPM1*, *CEBPA*, *IDH1*, WT1
Event of Interest	Mortality, early death, survival, prognosis
Language	English, Portuguese, Spanish
Study Types	Included: Randomized controlled trial, clinical trial, randomized trial<br>Not included: Review, meta-analysis, comment, editorial, letter, case reports

PubMed

PubMed (http://www.ncbi.nlm.nih.gov/pubmed) allows the development of advanced search terms. Combinations of search terms are given in supplementary Table S2.

Table S2. Search terms used for the PubMed database.

Element	Research Term
Participants	(Leukemia, Promyelocytic, Acute/mortality[MeSH] OR Leukemia, Promyelocytic, Acute/therapy[MeSH] OR "Acute Promyelocytic Leukemia" OR "AML M3" OR "Acute Myeloid Leukemia M3")
Exposure	AND<br>(FLT3 OR "Internal tandem duplications" OR ITD OR D835 ORRAS OR NPM1 OR CEBPA OR IDH1 OR TKD OR TKI OR "tyrosine kinase" OR "Wilms tumor 1" OR WT1 OR "Acquired mutation" OR "secondary mutation" OR "somatic mutation" OR "somatic change")
Interest Event	AND<br>(mortality OR death OR survival OR prognostic OR prognosis)
Language	AND<br>("english"[Language] OR "portuguese"[Language] OR "spanish"[Language])
Study Types Selection	NOT: (review[Title] OR review[Publication Type] OR "case reports"[Publication Type] OR "case reports"[Publication Type] OR editorial[Publication Type] OR letter[Publication Type] OR comment[Publication Type])

Cochrane

The search terms were initially used separately, and combined into a single search term in the last search step. The strategy is saved in the link: http://onlinelibrary.wiley.com/cochranelibrary/search/advanced/shared/searches/11369131229913378817

Table S3. Search terms used for the Cochrane Central Register of Controlled Trials (CENTRAL) database.

MeSH descriptor: [Leukemia, Promyelocytic, Acute] explode all trees and with qualifier(s): [Mortality - MO]	#1
"acute promyelocytic leukemia" or "AML M3" or "Acute Myeloid Leukemia M3"	#2
FLT3 or "Internal tandem duplications" or ITD OR D835 or RAS or NPM1 or CEBPA or IDH1 or TKD or TKI or “tyrosine kinase” or "Wilms tumor 1" or WT1 or "Acquired mutation" or "secondary mutation" or "somatic mutation" or "somatic change" or "secondary genetic disorder"	#3
Mortality or death or survival or prognostic or prognosis	#4
(#1 or #2) and #3 and #4	

Science Direct

The advanced search terms used in Science Direct (http://www.sciencedirect.com/) are shown in Table 5.

Table S4. Search terms used for the Science Direct database.

Element	Research Term
Participants	(Leukemia, Promyelocytic, Acute/mortality[MeSH] OR Leukemia, Promyelocytic, Acute/therapy[MeSH] OR "acute promyelocytic leukemia" OR "AML M3" OR "Acute Myeloid Leukemia M3")
Exposure	AND<br>TITLE-ABSTR-KEY(FLT3 OR "Internal tandem duplications" OR ITD OR D835 OR RAS OR NPM1 OR CEBPA OR IDH1 OR TKD OR TKI OR “tyrosine kinase” OR "Wilms tumor 1" OR WT1 OR "Acquired mutation" OR "secondary mutation" OR "somatic mutation" OR "somatic change" OR "secondary genetic disorder")
Interest Event	AND<br>TITLE-ABSTR-KEY(mortality OR death OR survival OR prognostic OR prognosis)
Study Types Selection	AND<br>LIMIT-TO(contenttype, "JL,BS","Journal") AND NOT TITLE(Review) AND NOT TITLE(meta-analysis) AND NOT TITLE(case report)

Scopus

Scopus (http://www.scopus.com) allows the user to build an advanced search term.

Table S5. Search terms used to query the Scopus database.

Element	Research Term
Participants	ALL ("acute promyelocytic leukemia" OR "AML M3" OR "acute promyelocytic leukemia")
Exposure	AND<br>TITLE-ABS-KEY(FLT3 OR "Internal tandem duplications" OR ITD OR D835 OR RAS OR NPM1 OR CEBPA OR IDH1 OR TKD OR TKI OR “tyrosine kinase” OR "Wilms tumor 1" OR WT1 OR "Acquired mutation" OR "secondary mutation" OR "somatic mutation" OR "somatic change" OR "secondary genetic disorder")
Interest Event	AND<br>TITLE-ABS-KEY((mortality OR death OR survival OR prognostic OR prognosis))
Language	AND<br>(LIMIT-TO(LANGUAGE,"English") OR LIMIT-TO(LANGUAGE,"Spanish") OR LIMIT-TO(LANGUAGE,"Portuguese"))

Study Types Selection	AND NOT TITLE(Review)<br>AND NOT TITLE(meta-analysis)<br>AND NOT TITLE(case report) AND (LIMIT-TO(SRCTYPE,"j")) AND SUBJAREA(MULT OR AGRI OR BIOC OR IMMU OR NEUR OR PHAR OR MULT OR MEDI OR NURS OR VETE OR DENT OR HEAL) AND (LIMIT-TO(DOCTYPE,"ar")

VHL

The VHL (http://pesquisa.bvsalud.org/) is a portal that allows the user to access MEDLINE, LILACS, and several other databases.

Table S6. Search terms used for the Virtual Health Library (VHL) database.

**Element**	**Research Term**
Participants	(tw:("acute promyelocytic leukemia" OR "AML M3" OR "Acute Myeloid Leukemia M3"))
Exposure	AND<br>(tw:(FLT3 OR "Internal tandem duplications" OR ITD OR D835 OR RAS OR NPM1 OR CEBPA OR IDH1 OR TKD OR TKI OR "tyrosine kinase" OR "Wilms tumor 1" OR WT1 OR "Acquired mutation" OR "secondary mutation" OR "somatic mutation" OR "somatic change" OR "secondary genetic disorder"))
Interest Event	AND<br>(tw:(mortality OR death OR survival OR prognostic OR prognosis))
Language	AND<br>(la:("en" OR "es"))
Study types Selection	AND (type:("article"))<br>AND NOT (TI:("case report" or "Review" or "apoptosis" or "meta-analysis"))

© 2019 by the authors. Licensee MDPI, Basel, Switzerland. This article is an open access article distributed under the terms and conditions of the Creative Commons Attribution (CC BY) license (http://creativecommons.org/licenses/by/4.0/).
